# Supplementary material for: The potential impact, cost and cost-effectiveness of tuberculosis interventions - a modelling exercise
Source: medRxiv. 2025 Sep 4:2025.09.02.25334943. Preprint. [Version 1] doi: 10.1101/2025.09.02.25334943 (PMC12424940; doi:10.1101/2025.09.02.25334943)
Supplement: Supplement 4 [file media-4.pdf]

SUPPLEMENTARY MATERIAL - S4 - RESULTS:
Comparing the relative impact, costs, and cost-effectiveness of TB interventions across three countries

Katherine C. Horton [1 2], Alvaro Schwalb [1 2 3], Martin J. Harker [1 2 4], Lara Goscé [1 2], Elena Venero-Garcia [1 2], Lily O'Brien [1 2], Arkaprabha Gun [1 2 5], Tom Sumner [1 2], C. Finn McQuaid [1 2], Rebecca A. Clark [1 2], Tomos O. Prys-Jones [1 2], Roel Bakker [1 2 6], Yiran E Liu [7 8], Mmamapudi Kubjane [9], Christian Lienhardt [10 11], Richard G. White [1 2], Rein M.G.J. Houben [1 2]

1 - TB Modelling Group, TB Centre, LSHTM, London, UK; 2 - Department of Infectious Disease Epidemiology, LSHTM, London, UK; 3 - Instituto de Medicina Tropical Alexander von Humboldt, Universidad Peruana Cayetano Heredia, Lima, Peru; 4 - Global Health Economics Centre, LSHTM, London, UK; 5 - Department of Epidemiology, Biostatistics, and Occupational Health, School of Population and Global Health, McGill University, Montreal, QC, Canada; 6 – KNCV Tuberculosis Foundation, The Hague, Netherlands; 7 - SEICHE Center for Health and Justice, Yale University School of Medicine, New Haven, CT, USA; 8 - Justice Collaboratory, Yale Law School, New Haven, CT, USA; 9 - Health Economics and Epidemiology Research Office, Wits Health Consortium, Johannesburg, South Africa; 10 - French Institute for Research in Sustainable Development (IRD), Montpellier, France; 11 - CRDF Global, Arlington, VA, USA

Correspondence: Rein M.G.J Houben, Department of Infectious Disease Epidemiology, London School of Hygiene and Tropical Medicine, Keppel St, London, WC1E 7HT United Kingdom (rein.houben@lshtm.ac.uk)

GitHub: https://github.com/lshtm-tbmg/NIH2

TABLE OF CONTENTS:

OVERVIEW.......... 2
1. CALIBRATION PLOTS AND POSTERIORS.......... 3
    1.1 Calibration plots.......... 4
        1.1.1 Brazil Calibration results.......... 4
        1.1.2 India Calibration results.......... 5
        1.1.3 South Africa Calibration results.......... 6
    1.2 Calibration posteriors.......... 7
2. EPIDEMIOLOGICAL IMPACT.......... 8
    2.1 Prevalence trends.......... 8
    2.2 Mortality trends.......... 9
    2.3 Incident sTB episodes averted - proportion of total.......... 10
    2.4 Incident sTB episodes averted - absolute numbers (1000s).......... 11
    2.5 Reduction in TB prevalence in 2050 - proportional (graph).......... 12
    2.6 Reduction in TB prevalence in 2050 - proportional (table).......... 13
    2.7 TB deaths averted - proportion of total.......... 14
    2.8 TB deaths - absolute numbers (1000s).......... 15
    2.9 Disability Adjusted Life Years averted - total (millions).......... 16
    2.10 Disability Adjusted Life Years averted - compared to BAU (millions).......... 17
3. ECONOMIC IMPACT.......... 18
    3.1 Total cost of each intervention.......... 18
    3.2 Incremental cost of each intervention compared to BAU.......... 19
    3.3 Incremental cost-effectiveness ratios (ICERs).......... 20
    3.4 Summary of economic results by country.......... 21
        3.4a Brazil.......... 21
        3.4b India.......... 23

3.4c South Africa....25
4. Sensitivity analyses....27
4.1 Aligned regression and progression parameters between India and South Africa.... 27
4.2 Aligned household size.... 28

OVERVIEW

This document provides additional results to those reported in the main paper. Throughout, we report median and 95% quantilles for the uncertainty intervals, except for the ICERS, where we use mean differences (section 3.3-3.4).

1. CALIBRATION PLOTS AND POSTERIORS

See supplementary materials S1, section 7, for calibration targets, data sources and methods.

1.1 Calibration plots

1.1.1 Brazil Calibration results

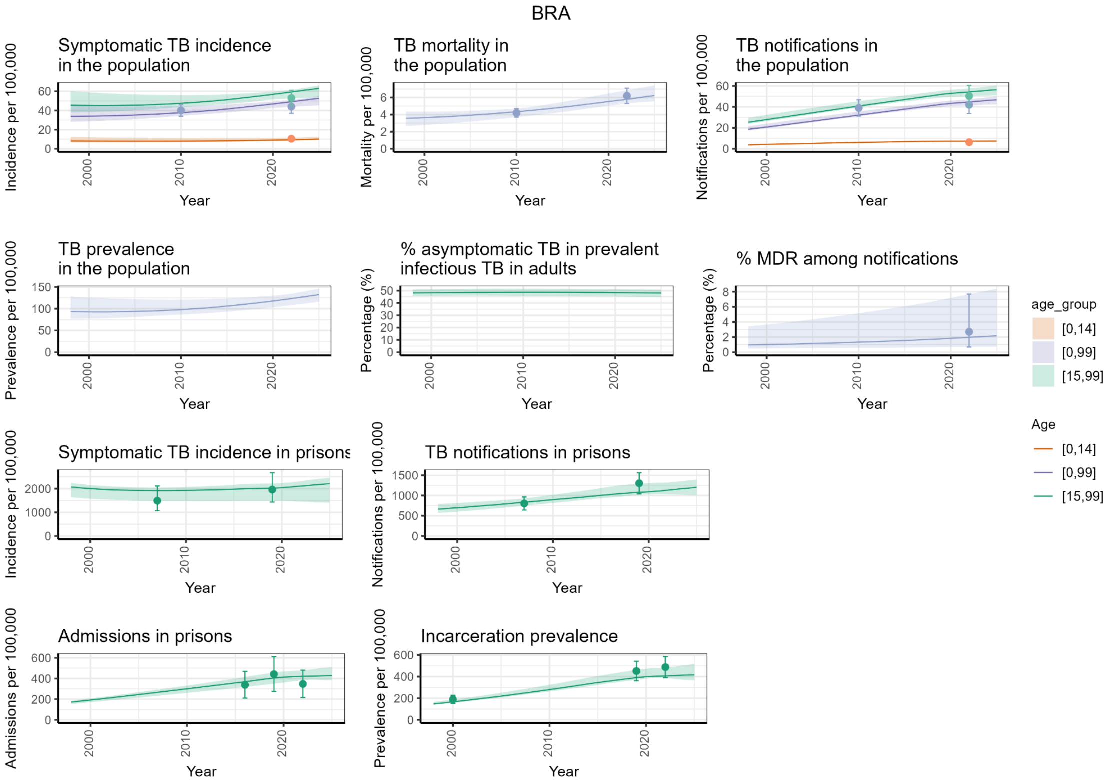

1.1.2 India Calibration results

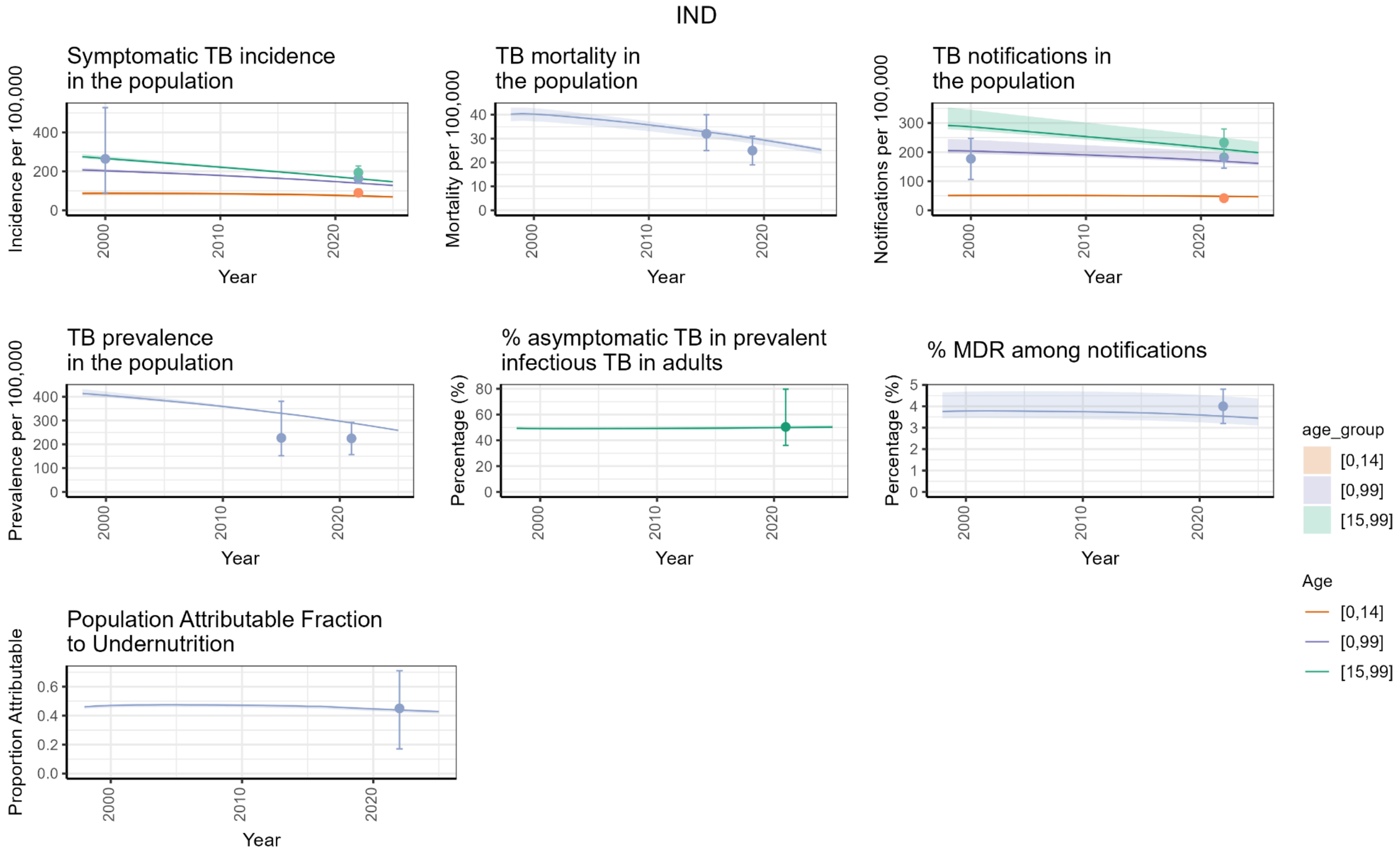

1.1.3 South Africa Calibration results

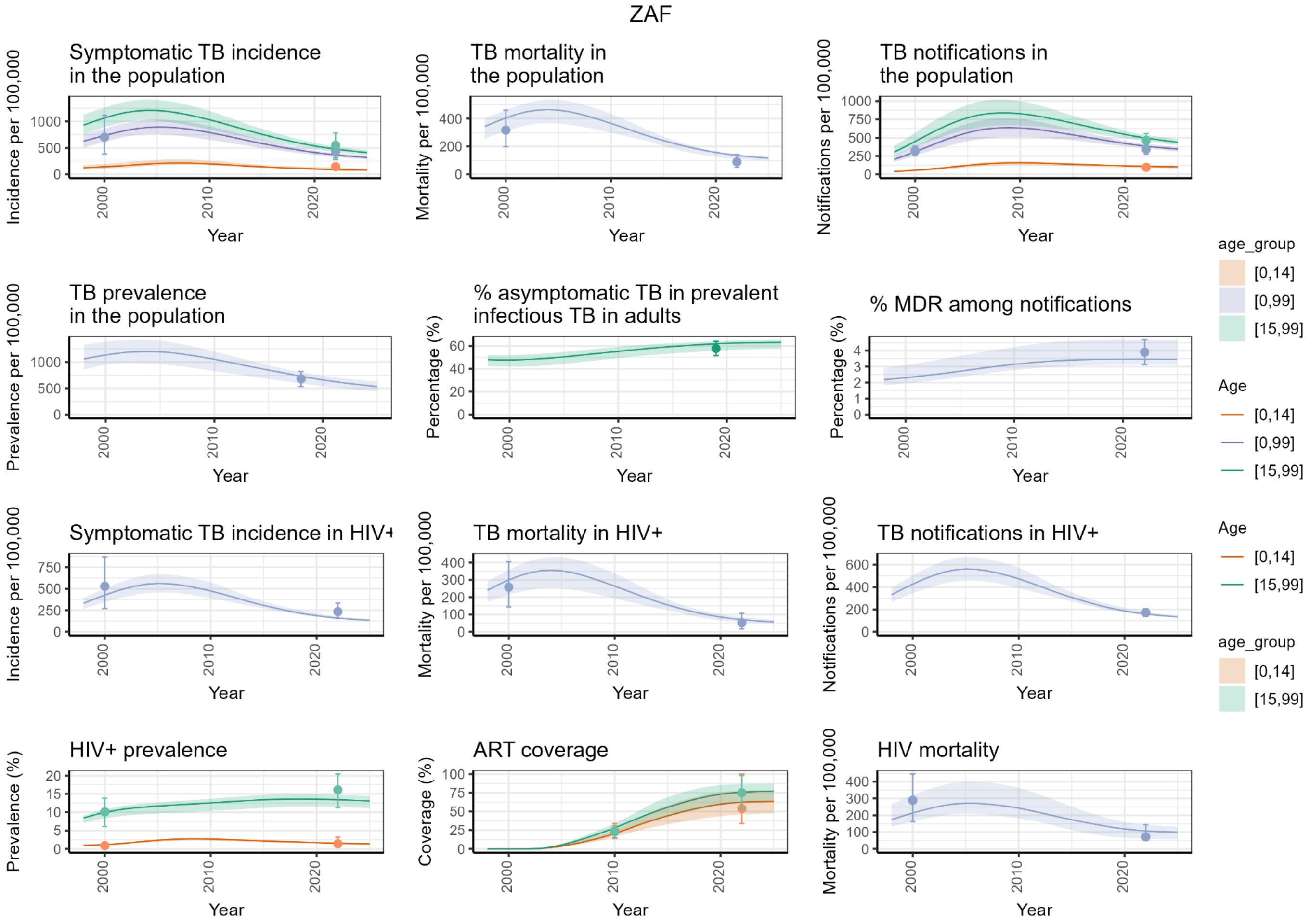

1.2 Calibration posteriors

Figures posterior distributions of parameters for each country (BRA = Brazil, IND = India, ZAF = South Africa). See supplementary materials S1 for parameters and prior ranges

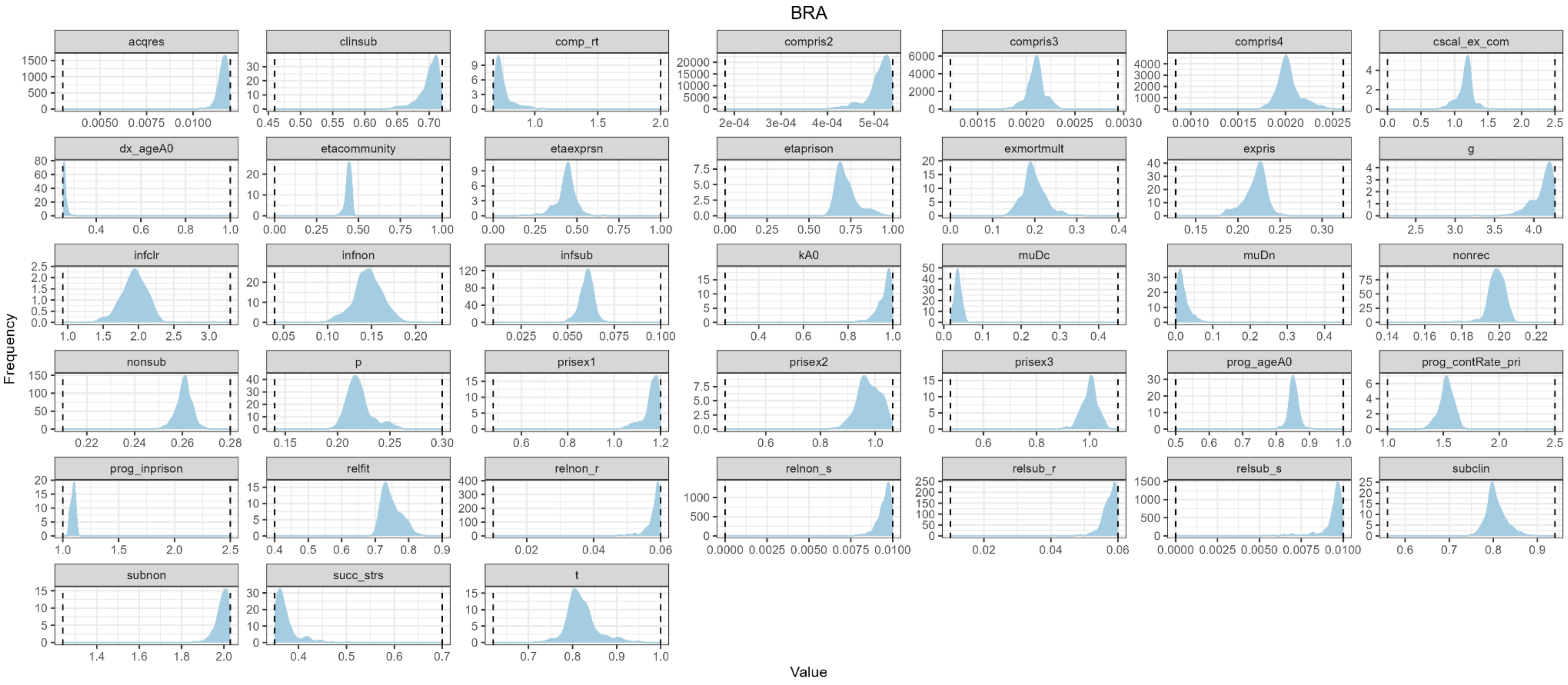

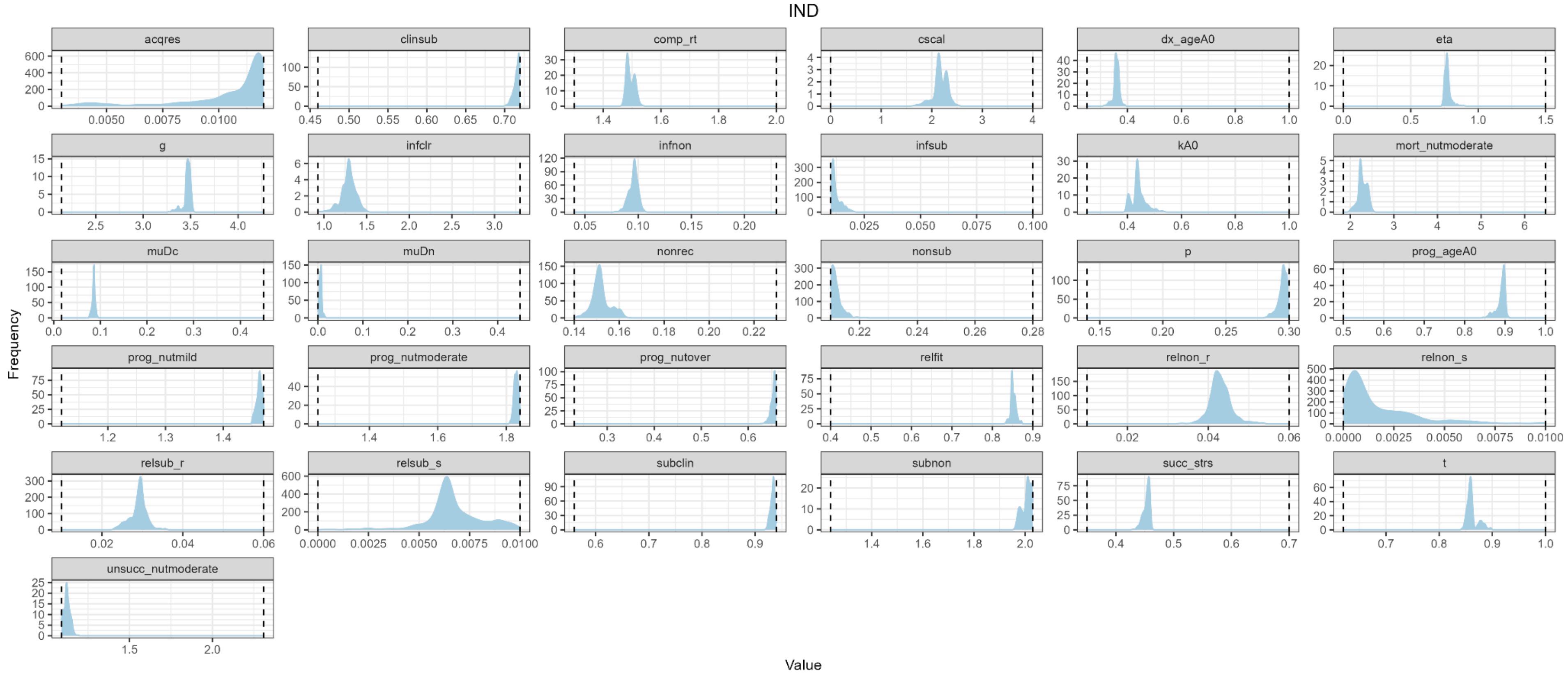
IND
acqres
clinsub
comp_rt
cscal
dx_ageA0
eta
g
infclr
infnon
infsub
kA0
mort_nutmoderate
muDc
muDn
nonrec
nonsub
p
prog_ageA0
prog_nutmild
prog_nutmoderate
prog_nutover
relfit
relnon_r
relnon_s
relsub_r
relsub_s
subclin
subnon
succ_strs
t
unsucc_nutmoderate
Frequency
Value

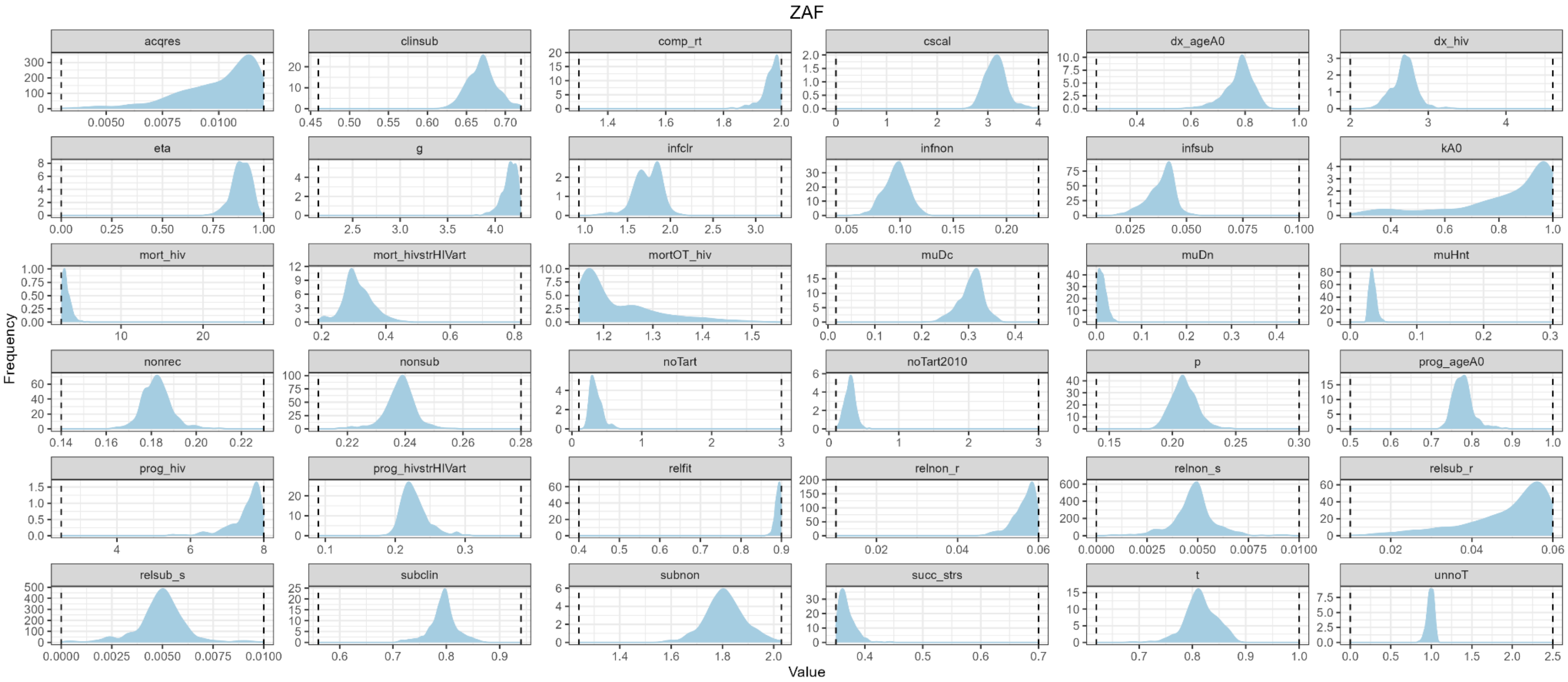
ZAF
acqres
clinsub
comp_rt
cscal
dx_ageA0
dx_hiv
eta
g
infclr
infnon
infsub
kA0
mort_hiv
mort_hivstrHIVart
mortOT_hiv
muDc
muDn
muHnt
nonrec
nonsub
noTart
noTart2010
p
prog_ageA0
prog_hiv
prog_hivstrHIVart
relfit
relnon_r
relnon_s
relsub_r
relsub_s
subclin
subnon
succ_strs
t
unnoT
Frequency
Value

2. EPIDEMIOLOGICAL IMPACT

2.1 TB Prevalence trends

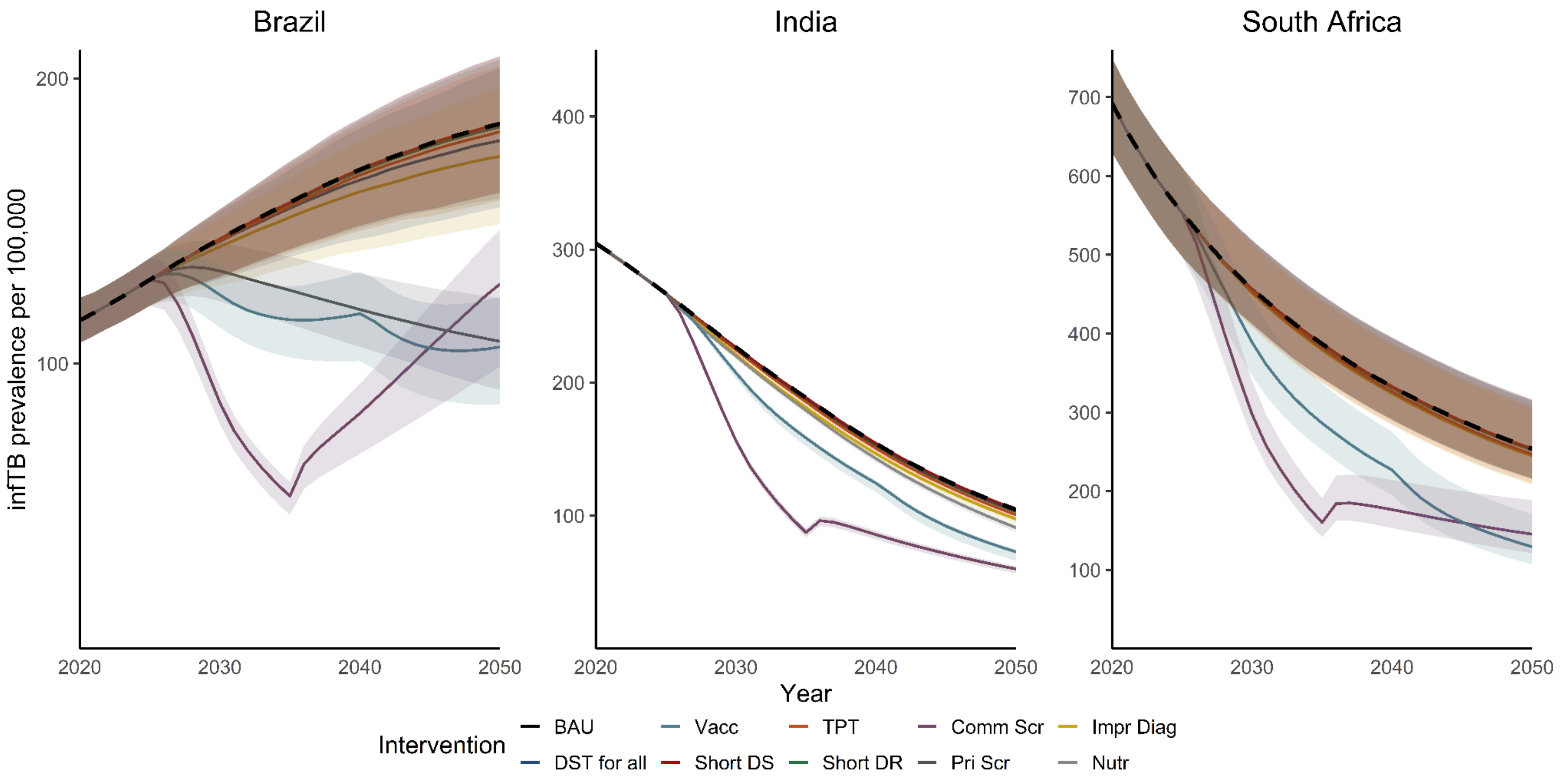

2.2 TB Mortality trends

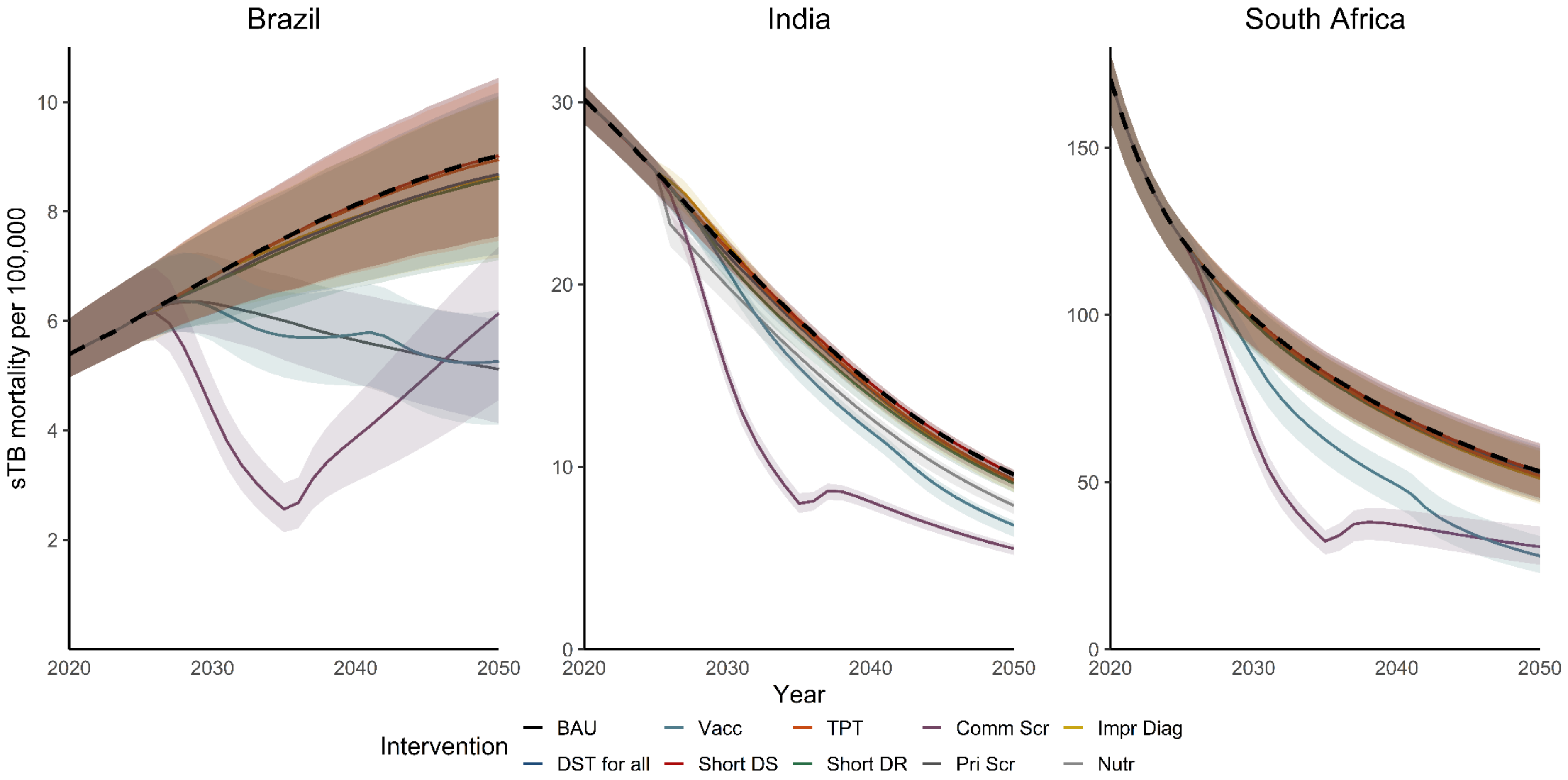

2.3 Incident sTB episodes averted - proportion of total

Incident symptomatic TB episodes averted by 2050 (percent relative to BAU)			
Intervention	Brazil	India	South Africa
Vacc	27.9% (26.6-30.2%)	14.8% (14.3-17.1%)	25% (22.6-28%)
TPT	0.8% (0.7-0.9%)	1.3% (1.3-1.4%)	1.1% (1-1.2%)
Comm Scr	38% (35.8-42%)	31.7% (30.5-32.8%)	35.5% (34.3-37.5%)
Impr Diag	2.4% (1.8-6%)	2.2% (2-2.6%)	1.3% (1.2-1.5%)
DST for all	0.8% (0.2-4%)	0.4% (0.3-0.5%)	0.1% (0.1-0.1%)
Short DS	0% (0-0%)	0% (0-0%)	0% (0-0%)
Short DR	0.1% (0-0.7%)	0.3% (0.2-0.3%)	0.2% (0.1-0.2%)
Pri Scr	23.3% (22-25.2%)	-	-
Nutr	-	5.1% (4.9-5.5%)	-

2.4 Incident sTB episodes averted - absolute numbers (1000s)

Incident symptomatic TB episodes averted by 2050 (number relative to BAU, in thousands)			
Intervention	Brazil	India	South Africa
Vacc	1,226K (1,086-1,333K)	6,038K (5,738-6,895K)	1,112K (963-1,328K)
TPT	34K (29-39K)	533K (525-562K)	49K (44-54K)
Comm Scr	1,662K (1,536-1,821K)	12,860K (12,419-13,182K)	1,585K (1,432-1,792K)
Impr Diag	103K (74-262K)	880K (816-1,052K)	60K (51-71K)
DST for all	34K (7-175K)	146K (126-205K)	4K (3-6K)
Short DS	0K (0-0K)	0K (0-0K)	0K (0-0K)
Short DR	6K (2-29K)	105K (92-138K)	7K (5-11K)
Pri Scr	1,022K (870-1,151K)	-	-
Nutr	-	2,050K (2,006-2,255K)	-

2.5 Reduction in TB prevalence in 2050 - proportional (graph)

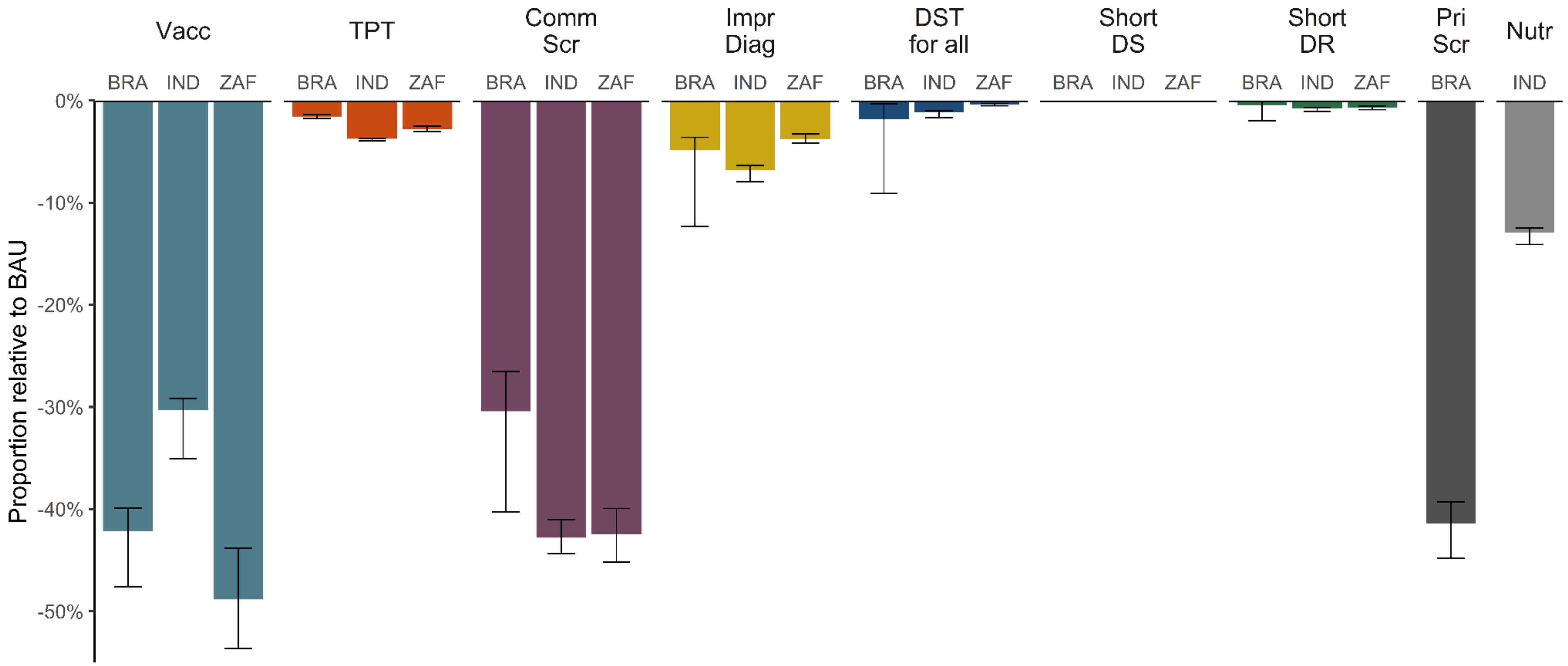

2.6 Reduction in TB prevalence in 2050 - proportional (table)

Decline in infectious TB prevalence in 2050 (percent relative to BAU)			
Intervention	Brazil	India	South Africa
Vacc	42.2% (39.9-47.6%)	30.3% (29.2-35%)	48.8% (43.8-53.7%)
TPT	1.6% (1.4-1.8%)	3.8% (3.7-4%)	2.8% (2.5-3%)
Comm Scr	30.4% (26.5-40.3%)	42.8% (41-44.4%)	42.5% (39.9-45.2%)
Impr Diag	4.9% (3.6-12.3%)	6.8% (6.4-7.9%)	3.8% (3.2-4.2%)
DST for all	1.8% (0.3-9.1%)	1.2% (1-1.7%)	0.4% (0.3-0.5%)
Short DS	0% (0-0%)	0% (0-0%)	0% (0-0%)
Short DR	0.4% (0.1-2%)	0.8% (0.7-1.1%)	0.7% (0.6-0.9%)
Pri Scr	41.4% (39.3-44.8%)	-	-
Nutr	-	12.9% (12.5-14.1%)	-

2.7 TB deaths averted - proportion of total

TB deaths averted by 2050 (percent relative to BAU)			
Intervention	Brazil	India	South Africa
Vacc	25.6% (24.2-28%)	12.8% (12.3-14.9%)	23.6% (21.1-26.5%)
TPT	0.4% (0.3-0.5%)	1.2% (1.2-1.2%)	1.1% (1-1.2%)
Comm Scr	42.2% (40.3-45.9%)	35.3% (33.4-36.3%)	38.1% (37-40%)
Impr Diag	1.8% (0.8-5.6%)	1.2% (0.9-1.4%)	1.8% (1.6-2%)
DST for all	1.9% (0.4-7.2%)	1.8% (1.5-2.3%)	0.8% (0.7-1.1%)
Short DS	0% (0-0%)	0% (0-0%)	0% (0-0%)
Short DR	2.5% (0.7-8.6%)	3.5% (3.1-4.6%)	1.7% (1.5-2.3%)
Pri Scr	25.2% (23.2-27.1%)	-	-
Nutr	-	11.1% (10.7-11.9%)	-

2.8 TB deaths - absolute numbers (1000s)

TB deaths averted by 2050 (number relative to BAU, in thousands)			
Intervention	Brazil	India	South Africa
Vacc	117K (102-135K)	871K (802-1,004K)	324K (279-381K)
TPT	2K (1-3K)	81K (77-84K)	15K (14-17K)
Comm Scr	194K (174-217K)	2,392K (2,238-2,458K)	528K (476-578K)
Impr Diag	8K (3-26K)	80K (61-93K)	26K (22-28K)
DST for all	9K (2-33K)	120K (104-159K)	11K (9-15K)
Short DS	0K (0-0K)	0K (0-0K)	0K (0-0K)
Short DR	11K (3-39K)	239K (209-304K)	24K (20-32K)
Pri Scr	115K (97-135K)	-	-
Nutr	-	749K (727-798K)	-

2.9 Disability-adjusted life years - total (millions)

Disability-adjusted life years (number)			
Intervention	Brazil	India	South Africa
BAU	10.8M (9.5-12.1M)	137.7M (132.6-140.6M)	23M (20.7-25M)
Vacc	7.9M (6.8-8.9M)	118.2M (112.4-121.3M)	17.2M (15.3-18.8M)
TPT	10.8M (9.4-12M)	135.9M (130.8-138.8M)	22.7M (20.4-24.7M)
Comm Scr	6.3M (5.2-7.1M)	88.7M (84.6-92.6M)	13.9M (12.3-15.4M)
Impr Diag	10.6M (9.1-11.8M)	135.5M (130.6-138.5M)	22.6M (20.3-24.5M)
DST for all	10.6M (9.2-11.9M)	135.7M (130.6-138.8M)	22.8M (20.5-24.8M)
Short DS	10.8M (9.5-12.1M)	137.7M (132.6-140.6M)	23M (20.7-25M)
Short DR	10.6M (9.2-11.9M)	133.9M (128.7-137.1M)	22.7M (20.4-24.6M)
Pri Scr	8.2M (7.2-9.2M)	-	-
Nutr	-	124.1M (119.1-127M)	-

2.10 Disability-adjusted life years averted compared to BAU (millions)

Disability-adjusted life years averted (number relative to BAU)			
Intervention	Brazil	India	South Africa
Vacc	2.9M (2.6-3.2M)	19.3M (18.1-22.2M)	5.7M (4.9-6.7M)
TPT	0.1M (0-0.1M)	1.8M (1.7-1.8M)	0.3M (0.2-0.3M)
Comm Scr	4.5M (4.1-5M)	48.9M (46.5-50.3M)	9M (8.2-9.9M)
DST for all	0.2M (0-0.7M)	1.9M (1.7-2.6M)	0.2M (0.1-0.2M)
Short DS	0M (0-0M)	0M (0-0M)	0M (0-0M)
Short DR	0.2M (0-0.6M)	3.6M (3.2-4.6M)	0.3M (0.3-0.5M)
Pri Scr	2.6M (2.2-3M)	-	-
Nutr	-	13.4M (13-14.4M)	-

3. ECONOMIC IMPACT

3.1 Total cost of each intervention

	Budget impact (USD)		
Intervention	Brazil	India	South Africa
BAU	1.6B (1.0-2.4B)	17.5B (12.3-25.6B)	2.5B (1.7-3.6B)
Vacc	4.0B (2.6-5.5B)	33.4B (23.8-44.6B)	3.1B (2.2-4.2B)
TPT	1.9B (1.2-3.0B)	20.4B (15.0-28.7B)	3.0B (2.1-4.1B)
Comm Scr (high)	37.6B (22.6-60.4B)	259.5B (165.7-370.2B)	12.0B (8.5-17.5B)
Comm Scr (low)	8.0B (5.7-10.8B)	49.0B (36.6-63.7B)	4.5B (3.4-5.8B)
Impr Diag	2.1B (1.4-3.4B)	29.0B (20.4-43.2B)	2.7B (1.9-3.7B)
DST for all	1.8B (1.1-2.9B)	18.3B (13.0-26.5B)	2.5B (1.7-3.6B)
Short DS	2.9B (1.9-4.3B)	32.5B (22.4-46.8B)	4.4B (2.9-6.3B)
Short DR	1.3B (0.8-1.9B)	17.2B (12.0-25.2B)	2.5B (1.6-3.5B)
Pri Scr	1.8B (1.2-2.6B)	-	-
Nutr	-	19.7B (14.5-28.0B)	-

3.2 Incremental cost of each intervention compared to BAU

	Incremental cost (USD relative to BAU)		
Intervention	Brazil	India	South Africa
Vacc	2.3B (1.3-3.8B)	15.8B (8.9-25.4B)	0.6B (0.3-1.0B)
TPT	0.3B (0.2-0.8B)	2.9B (2.1-4.2B)	0.5B (0.3-0.7B)
Comm Scr (high)	35.8B (20.8-58.8B)	241.5B (146.9-353.6B)	9.5B (6.0-14.9B)
Comm Scr (low)	6.4B (4.4-9.0B)	30.9B (21.4-42.5B)	1.9B (1.4-2.7B)
Impr Diag	0.5B (-0.2-1.4B)	11.2B (0.8-25.2B)	0.2B (-0.8-1.2B)
DST for all	0.2B (0.1-0.7B)	0.7B (0.5-1.2B)	0.0B (0.0-0.0B)
Short DS	1.3B (0.8-2.0B)	14.8B (9.3-22.0B)	1.9B (1.2-2.8B)
Short DR	-0.2B (-1.0--0.1B)	-0.3B (-0.6-0.0B)	0.0B (-0.1-0.0B)
Pri Scr	0.2B (0.1-0.3B)	-	-
Nutr	-	2.2B (1.5-3.1B)	-

3.3 Cost–utility analysis by country

ICERs are calculated based on the mean costs and mean DALYs across the 500 model runs, hence these figures differ slightly from the median values shown in tables 2.9, 2.10, 3.1 and 3.2 above. The probability of being cost-effective at the lower or upper end of the estimated cost-effectiveness threshold range is based on the proportion of the 500 model runs that produced results giving an ICER below the relevant threshold value. For more on how the threshold ranges were selected see *Supplementary Material S3*, section 2.6; these ranges are not official policy thresholds in the respective countries.

3.3a Brazil

**Intervention**	**Mean total cost** (billion R$)	**Mean total cost** (billion US$)	**Incremental cost compared to BAU** (million US$)	**Mean total DALYs** (million)	**DALYs averted compared to BAU** (million)	**ICER compared to BAU** (US$ per DALY averted)	**Probability cost-effective at lower end of threshold range**	**Probability cost-effective at upper end of threshold range**
Business as usual	8.04	1.61	-	10.80	-	-	-	-
Vaccination	19.84	3.98	2,365	7.87	2.93	807	100%	100%
TB preventive treatment	9.87	1.98	367	10.74	0.06	5,950	69%	85%
Community screening (high-cost)	192.29	38.54	36,924	6.29	4.52	8,174	31%	87%
Community screening (low-cost)	40.42	8.10	6,490	6.29	4.52	1,437	100%	100%

Improved diagnosis	10.89	2.18	572	10.52	0.28	2,040	97%	100%
Drug susceptibility testing for all	9.27	1.86	247	10.58	0.22	1,103	100%	100%
Shorter DS-TB treatment	14.84	2.97	1,363	10.80	0	Dominated by BAU	0%	0%
Shorter DR-TB treatment	6.36	1.27	-336	10.58	0.22	Dominates BAU	100%	100%
Screening in prisons	8.99	1.80	191	8.17	2.64	72	100%	100%

3.4b India

**Intervention**	**Mean total cost** (trillion INR)	**Mean total cost** (billion US$)	**Incremental cost compared to BAU** (billion US$)	**Mean total DALYs** (million)	**DALYs averted compared to BAU** (million)	**ICER compared to BAU** (US$ per DALY averted)	**Probability cost-effective at lower end of threshold range**	**Probability cost-effective at upper end of threshold range**
Business as usual	1.48	17.92	-	137.3	-	-	-	-
Vaccination	2.80	33.86	15.95	117.6	19.6	813	2%	12%
TB preventive treatment	1.73	20.89	2.98	135.5	1.8	1,658	0%	0%
Community screening (high-cost)	21.69	261.96	244.05	88.5	48.8	5,005	0%	0%
Community screening (low-cost)	4.06	49.09	31.17	88.5	48.8	639	1%	25%
Improved diagnosis	2.44	29.43	11.51	135.1	2.1	5,379	3%	3%
Drug susceptibility testing for all	1.55	18.68	0.76	135.3	2.0	390	60%	94%

Shorter DS-TB treatment	2.73	32.92	15.00	137.3	0	Dominated by BAU	0%	0%
Shorter DR-TB treatment	1.46	17.61	-0.31	133.6	3.7	Dominates BAU	100%	100%
Nutrition	1.67	20.17	2.25	123.8	13.5	167	100%	100%

3.4c South Africa

**Intervention**	**Mean total cost** (billion R)	**Mean total cost** (billion US$)	**Incremental cost compared to BAU** (million US$)	**Mean total DALYs** (million)	**DALYs averted compared to BAU** (million)	**ICER compared to BAU** (US$ per DALY averted)	**Probability cost-effective at lower end of threshold range**	**Probability cost-effective at upper end of threshold range**
Business as usual	47.22	2.56	-	22.94	-		-	-
Vaccination	57.95	3.14	582	17.18	5.76	101	100%	100%
TB preventive treatment	55.85	3.03	468	22.68	0.26	1,770	96%	100%
Community screening (high-cost)	227.73	12.34	9,784	13.91	9.03	1,083	100%	100%
Community screening (low-cost)	83.44	4.52	1,963	13.91	9.03	217	100%	100%
Improved diagnosis	50.46	2.74	176	22.52	0.42	420	93%	99%
Drug susceptibility testing for all	47.62	2.58	22	22.78	0.16	137	100%	100%

Shorter DS-TB treatment	82.72	4.48	1,924	22.94	0	Dominated by BAU	0%	0%
Shorter DR-TB treatment	46.53	2.52	-37	22.60	0.34	Dominates BAU	100%	100%

3.4 Cost-effectiveness planes for all interventions (including high-cost community-wide screening)

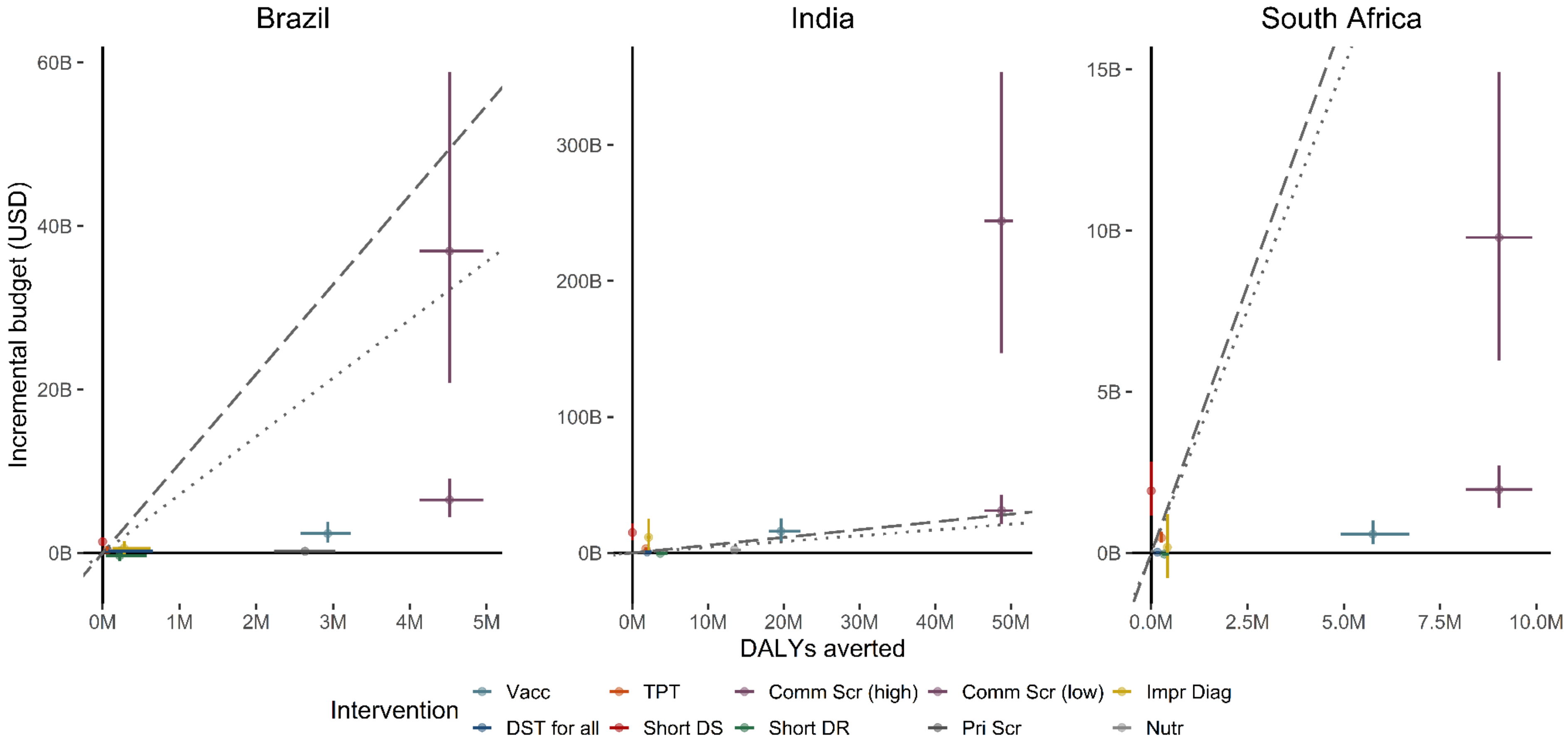

The dashed line shows the upper bound and the dotted line the lower bound of the estimated cost-effectiveness threshold range for each country. For a version of these graphs excluding high-cost community screening and showing the remaining interventions in more detail, see Figure 4 in the main paper.

4. Sensitivity analyses

4.1 Aligned regression and progression parameters between India and South Africa

We transferred the posterior parameters for TB natural history variables (*infsub, nonsub, nonrec and infclr*) for the South Africa calibration to India. While the calibration targets were not met, the proportional epidemiological impact for vaccination was now similar for India and South Africa.

Reduction in incident symptomatic TB in 2050		
	IND	ZAF
Vaccination	30.3% (29.1–35.0)	48.4% (43.3–53.2)
Sensitivity analysis	43.2% (NA–NA)	NA

4.2 Aligned household size

We applied the average household size for South Africa (2.3 adults per household; 0.9 children per household = 0.9) to India to look into the reason behind the different impact of TPT across countries, while the impact of community screening was similar. The proportional epidemiological impact for TPT in India decreased, suggesting that the higher impact of TPT in India compared to South Africa was due to differences in average household size.

Reduction in incident symptomatic TB in 2050		
	IND	ZAF
Community Screening	42.8% (41.0–44.3)	42.3% (39.7–45.0)
TPT	3.4% (3.3–3.5)	2.5% (2.3–2.7)
Sensitivity analysis	2.0% (NA–NA)	NA
